# Laparoscopic and Open Liver Resections for Colorectal Cancer Liver Metastasis in the Ukrainian State Center

**DOI:** 10.7759/cureus.38701

**Published:** 2023-05-08

**Authors:** Veronika Rozhkova, Anton Burlaka, Andrii Lukashenko, Yuriy Ostapenko, Volodymyr Bezverkhnyi

**Affiliations:** 1 Department of Minimally Invasive and Endoscopic Surgery, and Interventional Radiology, National Cancer Institute, Kyiv, UKR; 2 Department of Surgery, National Cancer Institute, Kyiv, UKR

**Keywords:** postoperative complication, oncological outcomes, colorectal cancer liver metastases, laparoscopic liver resection, open liver resection

## Abstract

Background

Minimally invasive liver resections for metastatic colorectal cancer have been increasingly performed all over the world with promising results. We planned the current study to review our experience on this matter and compare short- and long-term outcomes of laparoscopic liver resection (LLR) and open liver resection (OLR) in patients with colorectal cancer liver metastasis (CRLM).

Materials and methods

This is a single-center retrospective analysis of patients with CRLM who underwent laparoscopic (n=86) and open (n=96) surgical treatment for metastatic liver lesions between March 2016 and November 2022. Tumor characteristics, intra- and postoperative results, overall survival (OS), and disease-free survival (DFS) were analyzed and compared.

Results

LLR was associated with significantly shorter surgery duration (180 minutes versus 295 minutes, p=0.03). There was no significant difference in blood loss between the two groups (100 mL versus 350 mL, p=0.061). Additionally, the laparoscopic approach was associated with significantly shorter hospital stays (6 days versus 9 days, p=0.004). The rate of major complications (Clavien-Dindo classification ≥ 3) was lower in the LLR group (5.8% versus 16.6%, p=0.037). There was no mortality in the LLR group, and in the OLR group, one lethal case was induced by mesenteric thrombosis on the fifth postoperative day. We did not find a statistically significant difference in the OS rate between the two groups at one, three, and five years: 97.3%, 74.7%, and 43.4%, respectively, in the OLR group and 95.1%, 70.3%, and 49.5%, respectively, in the LLR group (p=0.53). DFS at one, three, and five years were 88.7%, 52.3%, and 25.5%, respectively, in the LLR group and 71.9%, 53.1%, and 19.3%, respectively, in the OLR group (p=0.66).

Conclusions

This study showed that laparoscopic liver surgery is a safe and effective method of CRLM treatment in our center. LLR was associated with a decrease in major morbidity, shorter surgery duration, and reduced postoperative hospital stay. Minimally invasive liver resections showed similar oncological outcomes to the open approach in terms of overall and disease-free survival.

## Introduction

In 2020, colorectal cancer was diagnosed in more than 1.9 million people, ranking third in terms of incidence and second in terms of cancer-related mortality, accounting for 9.4% of lethal cases [1]. In Ukraine, it is a big healthcare issue as well. According to the National Cancer Registry, 35.3% of patients develop liver metastasis within the first three years after the diagnosis of a primary tumor [2,3]. The main treatment option in such cases remains a radical liver resection, which allows to achieve five-year survival of 40%-60% [4].

Scientific evidence on the feasibility of minimally invasive techniques in the field of liver surgery is confined to retrospective series, meta-analysis, and results of randomized controlled trials (RCTs), such as the OSLO-COMET and LapOpHuva [5-7]. The obtained data seem to favor the laparoscopic approach in terms of reduction in complication rate and postoperative hospital stay, which made laparoscopic liver resection (LLR) a standard of care in many centers. Therefore, our center has adopted laparoscopic liver resections in the setting of the Ukrainian state center, facing challenges associated with this. We planned the current study to ensure that our results are feasible and adequate in the reality of our institution. The aim was to analyze surgical safety and oncological outcomes of LLR in patients with colorectal cancer liver metastases (CRLM).

## Materials and methods

Patient selection

We performed a retrospective analysis of colorectal cancer patients with liver metastasis (CRLM) who received laparoscopic (n=85) and open (n=96) surgical treatment for metastatic liver lesions between March 2016 and November 2022 at the National Cancer Institute.

The inclusion criterion was a curative intended resection directed toward the removal of all radiologically evident liver lesions. In total, 181 CRLM patients with morphologically confirmed adenocarcinoma of the colon or rectum and ≥1 liver metastasis were included in the study. We excluded patients < 18 years old, those who carried more than five resectable lung metastases, and/or unresectable peritoneal carcinomatosis.

The preoperative investigations were conducted according to international standards: blood tests, contrast-enhanced CT, and/or MRI. All patients were discussed at the multidisciplinary board meeting. Liver anatomy and resection were classified according to The Brisbane 2000 Terminology, based on Couinaud’s segments [8].

Short- and long-term outcomes

The clinical parameters examined were age, gender, American Society of Anesthesiologists (ASA) score, body mass index (BMI), and CRLM characteristics. Surgical parameters such as the type of resection, operation time, conversion rate, and blood loss were collected. Primary outcomes were assessed by postoperative morbidity, mortality, and length of hospital stay. Major complications were defined either as requiring intensive care unit stay, treatment by an interventional radiologist, or reoperation [9]. Long-term outcomes were estimated by overall survival (OS) and disease-free survival (DFS) after liver surgery.

Surgical technique

Surgical techniques included anatomical-oriented, parenchymal-sparing open liver resection (OLR) and laparoscopic liver resection (LLR), and radiofrequency ablation (RFA) for deeply located tumors ≤ 2 cm. Major hepatectomy was defined as the resection of three or more contiguous liver segments. All surgeries were performed under general anesthesia. To locate the tumor and guide the resection plane according to anatomical landmarks, we used intraoperative ultrasonography. The patient’s position and operative setup for laparoscopic resections were described in detail elsewhere [10,11]. Trocars were positioned based on the tumor’s location and the patient’s habitus. For transection, we used high-energy devices. For open liver resection, laparotomy was done via a right subcostal incision or J-shaped laparotomy extended to the right. Parenchymal transection was performed using the crash clamp technique or cavitron ultrasonic surgical aspirator (CUSA).

Statistics

For nonparametric data, to define the normality of distribution, the Kolmogorov-Smirnov test was used. Continuous variables were expressed as median or mean and range interval depending on the normality of distribution. To compare normally distributed data, the Mann-Whitney U test was used. For data with skewed distribution, Student’s t-test was applied. Categorical variables were reported as number (n) and percentage (%) and compared using the chi-squared test (χ^2^) (with Yates’ correction or not) and Fisher’s exact test. P-values of <0.05 were considered statistically significant. Survival was evaluated via the Kaplan-Meier method and compared using the log-rank test.

## Results

Baseline characteristics of patients

Overall, 181 patients who were operated between March 2016 and November 2022 met our inclusion criteria. Among those, 85 (46.9%) patients underwent liver resection via laparoscopic approach, and 96 (53%) underwent open liver resections (OLR). The primary characteristics of patients are presented in Table 1. The two groups were distributed similarly albeit the number of metastatic liver lesions, which was significantly higher in the open resection group.

**Table 1:** Demographic characteristics and preoperative variables ^a^By Mann-Whitney U test ^b^By Fisher’s exact test LLR: laparoscopic liver resection, OLR: open liver resection, SD: standard deviation, BMI: body mass index, ASA: American Society of Anesthesiologists, n: number

Values	LLR (n=85)	OLR (n=96)	P-value
Male:female	44:41	51:45	0.97^b^
Age (mean±SD)	55.9±10	60.3±11.3	0.75^a^
BMI, kg/m^2^ (mean, min-max)	27.8 (19.2-47.9)	24.1 (15.8-40.1)	0.68^a^
ASA score (I/II/III)	12/63/10	8/75/13	0.71^b^
Primary tumor localization			0.89^b^
Right-sided, % (n)	15.3% (13/85)	14.6% (14/96)	
Left-sided/rectum, % (n)	84.7% (72/85)	85.4% (82/96)	
Positive regional lymph nodes of the primary tumor, % (n)	49.9% (42/85)	40.6% (39/96)	0.24^b^
Synchronous status of liver metastases, % (n)	51.8% (44/85)	62.5% (60/96)	0.29^b^
Preoperative chemotherapy, % (n)	65% (55/85)	70.8% (68/96)	0.35^b^
Size of the biggest liver lesion, mm (median, min-max)	23 (6-60)	30 (10-150)	0.068^a^
Number of metastatic liver lesions (median, min-max)	1 (1-6)	2 (1-18)	0.001^a^
Bi-lobar liver metastasis	23.3% (20)	32.3% (31)	0.19^b^
Distribution based on localization in liver segments			0.076^b^
Posterosuperior segments (Sg1,4a,7,8)	46.7% (40/85)	58.3% (56/96)	
Anterolateral segments (Sg2,3,4b,5,6)	53.3% (45/85)	41.7% (40/96)	

Operative and postoperative data

**Figure 1:**
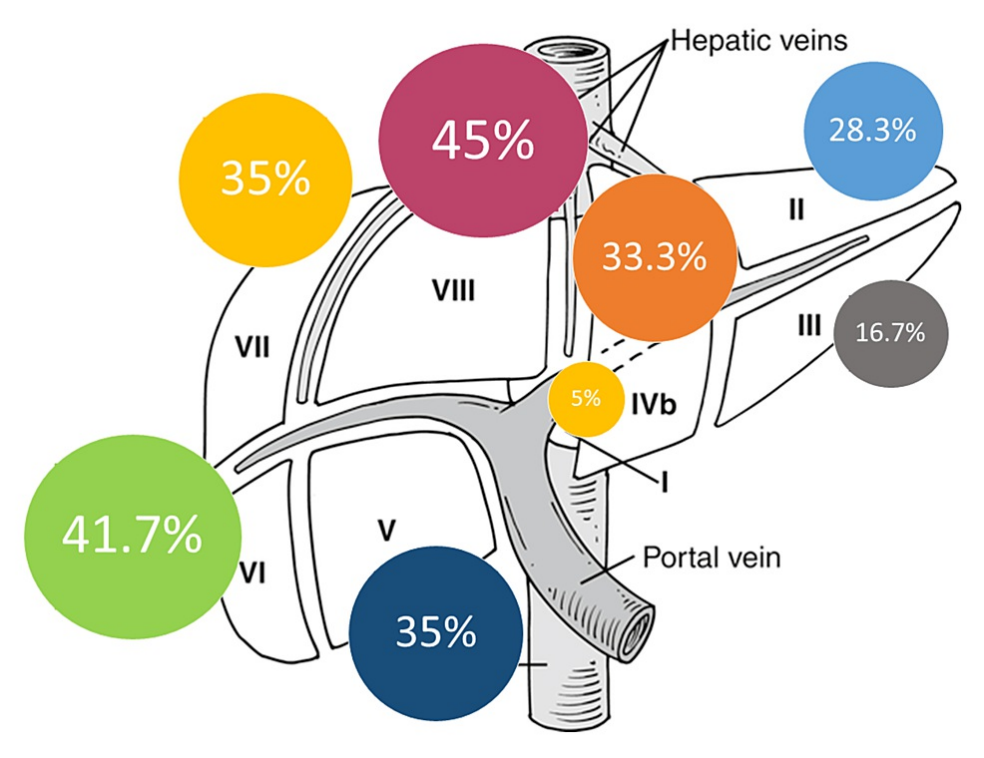
Frequency of liver segments’ resections in the LLR group Figure 1 is the author’s own creation and does not require permission to publish. LLR: laparoscopic liver resection

**Figure 2:**
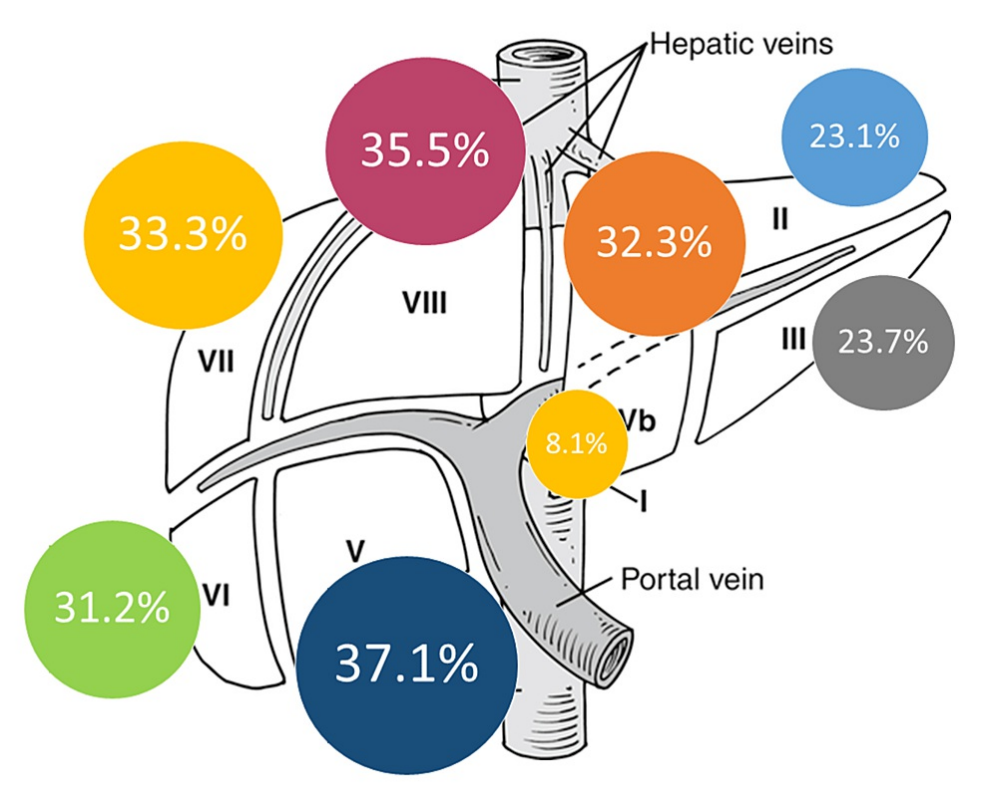
Frequency of liver segments’ resections in the OLR group Figure 2 is the author’s own creation and does not require permission to publish. OLR: open liver resection

Figure 1 and Figure 2 show the distribution of liver segments’ resections in both groups. In the LLR group, the most frequently resected segments were Sg8 and Sg6 (45% and 41.7%, respectively). In the OLR group, it was Sg5 and Sg8 (37.1% and 35.5%, respectively).

There was no difference in the amount of resected liver segments between the two groups (p=0.07). In the OLR group, 8.3% (8/96) of patients underwent major liver resections, and in the LLR group, it was 3.5% (3/85). In the laparoscopic liver resection group, we performed radiofrequency ablation (RFA) in 8.2% (7/85) of patients (Table 2).

**Table 2:** Surgery characteristics ^a^By Mann-Whitney U test ^b^By Fisher’s exact test ^c^By chi-squared test LLR: laparoscopic liver resection, OLR: open liver resection, RFA: radiofrequency ablation

Values	LLR (n=85)	OLR (n=96)	P-value
Number of resected liver segments (median, min-max)	2 (1-6)	3 (1-7)	0.079^a^
Type of liver resection			0.081^b^
Minor liver resection	96.5% (82/85)	91.7% (88/96)	
Major liver resection	3.5% (3/85)	8.3% (8/96)	
RFA	8.2% (7/85)	1% (1/96)	0.02^b^
Simultaneous surgery of the colon/rectum and liver	10.5% (9/85)	16.7% (16/96)	0.24^c^
Conversion rate	5.9% (5/85)	-	

Surgery duration was shorter in the laparoscopic liver resection group (180 minutes versus 295 minutes, p=0.03). In the LLR group, there were five conversions (5.9%) due to intra-abdominal adhesions after previous colon and/or liver resections and the inability to safely perform adhesiolysis via a laparoscopic approach.

As shown in Table 3, the laparoscopic approach was associated with significantly shorter hospital stays (6 days versus 9 days, p=0.004). Additionally, we found a significant difference in postoperative major morbidity rate, which was lower in the LLR group (5.8% versus 16.6%, p=0.037). The most frequent complications were bile leakage, biloma, and pleuritis that required puncture. There was no mortality in the LLR group, and in the OLR group, one lethal case was induced by mesenteric thrombosis on the fifth postoperative day.

**Table 3:** Intra- and postoperative variables ^a^By Mann-Whitney U test ^b^By Fisher’s exact test LLR: laparoscopic liver resection, OLR: open liver resection

Values	LLR (n=85)	OLR (n=96)	P-value
Surgery duration, minutes (median, min-max)	180 (45-455)	295 (35-735)	0.03^a^
Blood loss, mL (median, min-max)	100 (20-800)	350 (50-1500)	0.061^a^
Postoperative hospital stay, days (median, min-max)	6 (2-16)	9 (4-41)	0.004^a^
Major morbidity	5.8% (5/85)	16.6% (16/96)	0.037^b^
30-day mortality	0	1% (1/96)	0.69^b^

**Figure 3:**
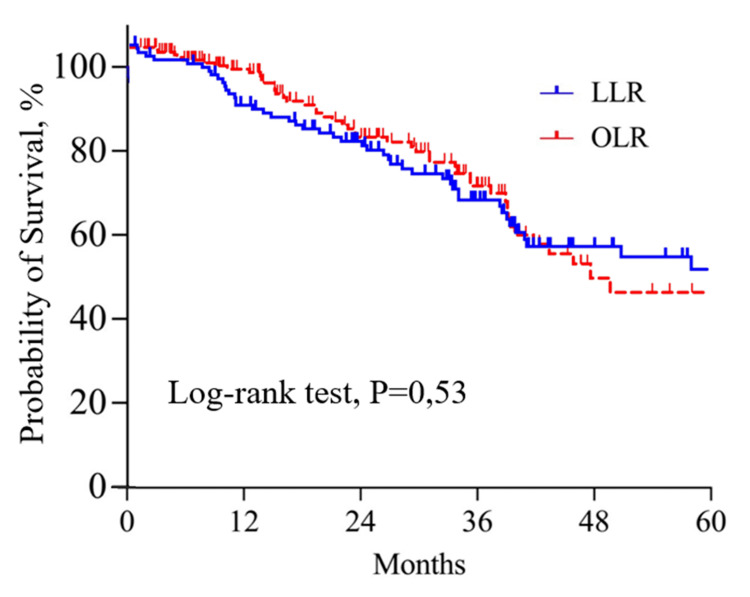
Overall survival in open and laparoscopic liver resection groups OLR: open liver resection group, LLR: laparoscopic liver resection group

**Figure 4:**
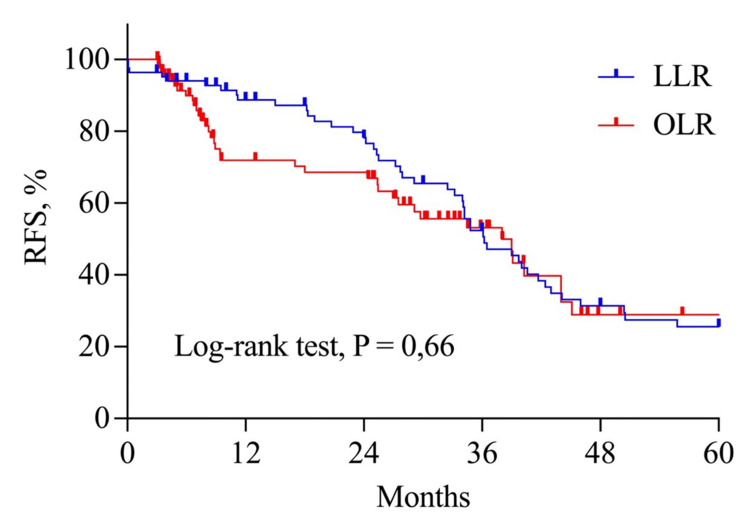
Disease-free survival in open and laparoscopic liver resection groups OLR: open liver resection group, LLR: laparoscopic liver resection group

The median survival was 36.2 months and 38 months in the LLR and OLR groups, respectively. As Figure 3 shows, the OS was similar between the two groups at one, three, and five years: 97.3%, 74.7%, and 43.4%, respectively, in the OLR group, and 95.1%, 70.3%, and 49.5%, respectively, in the LLR group (p=0.53). Disease-free survival at one, three, and five years were 88.7%, 52.3%, and 25.5%, respectively, in the LLR group and 71.9%, 53.1%, and 19.3%, respectively, in the OLR group (p=0.66) (Figure 4). Liver-only recurrence was found in 32.9% of patients in the LLR group and 39.1% in the OLR group (p=0.18).

## Discussion

This study compares the results of laparoscopic and open liver resections in patients with colorectal cancer liver metastasis who were treated in the settings of a Ukrainian state center. We report successful short- and long-term outcomes of laparoscopic liver resection in patients with CRLM.

A number of retrospective studies showed that laparoscopic liver resections are more favorable in terms of postoperative complication rate [12-15]. Others, however, did not find any significant difference in major morbidity between the two groups [16-18]. This contradiction could be resolved by the OSLO-COMET trial, where the LLR group had a reduced rate of postoperative complications (31% versus 19%) [19]. However, the authors noted that if the open group had only three fewer complications, the primary endpoint would lose its statistical significance. A systematic review and meta‑analysis by Ciria et al. (2020) [20] showed that in studies on minor-only or major-only laparoscopic versus open liver resections, there was no significant difference in complication rate. According to our results, the LLR group had lower major postoperative morbidity (5.8% versus 16.6%, p=0.037). Such a decrease in morbidity rate may beneficially affect overall survival as the occurrence of complications could delay the onset of adjuvant chemotherapy, which is a pivotal component in the treatment of CRLM [21].

As to the long-term outcomes, multiple studies have proven that the laparoscopic approach does not impair overall survival [7,22-24]. We concur with this data: the five-year overall survival in the LLR group was 49.5%, and in the OLR group, it was 43.4% (p=0.53). Interestingly, the meta-analysis by Syn et al. (2020) [25] that involved 3,148 patients from two RCTs and 13 propensity-score matched (PSM) studies demonstrated the survival benefit of the laparoscopic approach over open resection. They defined eight possible mechanisms to explain this finding. Those mainly come down to enhanced recovery after laparoscopy, reduction of surgical stress, and implementation of a parenchymal-sparing approach. Indeed, a parenchymal-sparing strategy in a setting of colorectal cancer liver metastasis is especially beneficial due to the high possibility of recurrence in the residual liver and the necessity for subsequent liver resections. Aghayan et al. (2017) [26] reported liver recurrence in 46% of patients after laparoscopic parenchymal-sparing liver surgery. Among them, 68% of cases were applicable for repeated curative liver resections. Martínez-Cecilia et al. (2021) [27], in their propensity score matching analysis, also found a statistically significant difference between repeated liver resections in open and laparoscopic groups (30% versus 48%, p<0.001). Furthermore, the laparoscopic approach facilitates further liver surgeries, especially repeat LLRs, because of the reduced formation of intra-abdominal adhesions [28].

Starting from the consensus conference held in Louisville, laparoscopic resections of posterosuperior liver segments (PSS) have been considered “major” from a technical point of view and were suggested to be performed at expert centers only [29]. Teo et al. (2015) [10] conducted a comparison study between the LLR of posterosuperior and anterolateral segments. There was higher blood loss in the PSS group, as well as conversion rate, the most common reason for which was uncontrollable bleeding. In our institution, localization of metastatic lesions in posterosuperior segments was not a contraindication for laparoscopy: there was no difference in the amount of PSS resections between open and laparoscopic groups.

Implementation of a highly specialized surgical project such as a laparoscopic liver resection program requires significant initial and sustained efforts [30]. Analysis of these six years’ experience in laparoscopic liver surgery helped us define several issues that we faced when launching this program in the settings of the Ukrainian state center. Firstly, the education of our surgical team was not systematic. Secondly, because of governmental underbudgeting, we do not possess sufficient technical equipment. Thirdly, there is patient hesitation due to unawareness of the benefits of minimally invasive approaches. These issues and their solutions will be the subject of our further research projects.

The present study has a number of limitations. Firstly, retrospective studies are prone to selection bias with regard to operative methods. Secondly, conclusions should be carefully withdrawn due to a rather small cohort of patients. Thirdly, since the data was collected from a single institution, there is a place for center-related bias. Additionally, the beginning of the study period was associated with a learning curve that could influence the results.

## Conclusions

Surgical resection remains the most radical treatment option for patients with colorectal cancer liver metastasis. A minimally invasive approach can be an advantageous strategy for such patients. We found that LLRs are associated with better short-term outcomes: decreased major morbidity, shorter surgery duration, and significantly lower postoperative hospital stay. As for oncological outcomes, LLRs are equivalent to open resections in terms of overall and disease-free survival. Therefore, laparoscopic liver resections are a feasible method of surgical treatment, safely adapted to the reality of our institution.
